# Circulating Tumor DNA (ctDNA) Dynamics Predict Early Response to Treatment in Metastasized Gastroesophageal Cancer (mGEC) After 2 Weeks of Systemic Treatment

**DOI:** 10.3390/cancers16233960

**Published:** 2024-11-26

**Authors:** Stefan Tatalovic, Bernhard Doleschal, Alexander Kupferthaler, Stephan Grundner, Jonathan Burghofer, Gerald Webersinke, Simon Schwendinger, Emina Jukic, Johannes Zschocke, Lorenz Danhel, Antonia Kirchweger, Lukas Havranek, Demetre Shalamberidze, Daniel Rezaie, Matthias Biebl, Holger Rumpold, Patrick Kirchweger

**Affiliations:** 1 Department of Surgery, Ordensklinikum Linz, 4010 Linz, Austria; stefan.tatalovic@ordensklinikum.at (S.T.);; 2 Medical Faculty, Johannes Kepler University Linz, 4020 Linz, Austria; 3 VYRAL, 4020 Linz, Austria; 4 Department of Internal Medicine I for Hematology with Stem Cell Transplantation, Hemostaseology and Medical Oncology, Ordensklinikum Linz, 4010 Linz, Austria; 5 Department of Diagnostic and Interventional Radiology, Ordensklinikum Linz, 4010 Linz, Austria; 6 Laboratory for Molecular Genetics Diagnostics, Ordensklinikum Linz, 4010 Linz, Austria; 7 Institute of Human Genetics, Medical University Innsbruck, 6020 Innsbruck, Austria

**Keywords:** ctDNA, gastric cancer, esophageal cancer, liquid biopsy

## Abstract

mGEC is associated with poor overall survival (OS) of approximately 4–10 months. CtDNA is emerging as a promising prognostic biomarker with high potential for early relapse detection. However, until now, there was little knowledge on serial ctDNA detection and its impact on early treatment evaluation and prognosis in mGEC. Methods: ctDNA detection (ddPCR) was carried out serially in 37 matched tissue (NGS) patients with mGEC prior to systemic treatment initiation and every two weeks thereafter until restaging (n = 173 samples). The results have been correlated with response to treatment (restaging CT), overall survival (OS), and progression-free survival (PFS). Results: The pretherapeutic detection rate was 77.8%. Response to treatment assessment was correct in 54.2% (pretherapeutically pos./neg.) and 85.7% (dynamics at week 4). Moreover, a decline in ctDNA (MAF in %) below 57.1% of the pretherapeutic value after 2 weeks of systemic treatment was accompanied by a sensitivity of 57.1% and a specificity of 90% (AUC = 0.73) for correct restaging assessment (response evaluation by CT after 3 months) evaluating 76.5% of patients correctly after only 2 weeks. In contrast to mere pretherapeutic ctDNA positivity (*p* = 0.445), a decline in ctDNA dynamics to under 57.1% of its initial value was significantly associated with OS (4.1 (95% Cl 2.1–6.1) vs. 13.6 (95% CI 10.4–16.6) months, *p* < 0.001) and PFS (3.2 (1.9–4.5) vs. 9.5 (95% CI 5.5–13.5) months, *p* = 0.001) after two weeks of treatment. Additionally, the change in detectability from positive pretherapeutic levels to negative during treatment was associated with similar survival as for patients who were always regarded as ctDNA-negative (9.5 (95%Cl 0.4–18.5) vs. 9.6 (95%Cl 1.3–17.9)). The absence of becoming undetectable was associated with worse survival (4.7 months). Conclusions: ctDNA is a promising additional biomarker allowing for early evaluation of response to treatment and saving unevaluated treatment time for patients with mGEC, and could allow for an early change in treatment with anticipated prognostic benefit in the future.

## Simple Summary

This pioneering study investigates the prognostic value of circulating tumor DNA (ctDNA) as a biomarker for monitoring treatment response in metastatic gastroesophageal cancer (mGEC). Despite advancements in personalized and multimodal treatments, the prognosis for mGEC remains poor, with low survival rates. ctDNA has emerged as a promising tool for non-invasive cancer monitoring, but its clinical implementation has not yet been fully realized. This study marks a pioneering effort in evaluating ctDNA kinetics using a serial liquid biopsy method that is easy to implement and clinically applicable. The study found that ctDNA changes within the first two weeks of chemotherapy were significantly associated with treatment response, with a 57.1% decline in ctDNA levels correlating with a better prognosis. This method was able to predict treatment response 80% faster than the current gold standard, computed tomography (CT), providing early insight into the effectiveness of systemic chemotherapy. Despite advances in personalized and multimodal treatment in recent years, the prognosis of metastasized gastroesophageal cancer (mGEC) is still poor. Circulating tumor DNA (ctDNA) has evolved as a promising new biomarker for treatment monitoring but has failed to reach clinical implementation yet. This study shows the immediate prognostic impact of ctDNA kinetics using an easily implementable clinical applicable method of serial liquid biopsies. Additionally, this personalized method enables early response to treatment evaluation 80% faster than current gold standard computed tomography (CT) after only 2 weeks of systemic chemotherapy.

## 1. Introduction

Gastroesophageal cancer (GEC) is the sixth most common cause of cancer-related death worldwide [1]. Incidence is increasing, with 604,000 new cases opposing 544,000 deaths worldwide from GEC in 2020 [2]. Despite substantial advancements in the management of GEC, the prognosis remains poor, with five-year survival rates under 20%. This high mortality rate is largely attributable to the advanced stage at which most patients are diagnosed, resulting in extensive disease progression [3]. For patients with distant metastatic disease (stage IV), the five-year survival rate plummets to 2–3% [4]. The ESMO Clinical Practice Guidelines for 2024 state that the most common toxicities are hematologic complications such as neutropenia and anemia, as well as gastrointestinal effects like esophagitis, nausea, and vomiting. It is essential to manage these adverse effects carefully and consider dose adjustments, as they can potentially compromise the efficacy of the treatment regimen [4,5,6]. Novel biomarkers such as ctDNA might have the potential to change clinical practice and could add valuable assistance to the management of various cancers disease [7]. Therefore, circulating DNA (ctDNA) has emerged as a popular tool in the diagnostics of cancer because it offers an easy non-invasive method to identify tumor-specific abnormalities (e.g., mutation, methylation). This offers many potential applications such as additional markers for diagnosis, follow-up of treatment, or estimation of prognosis [8]. The potential of ctDNA is not limited to one tumor entity but has already been evaluated in various types of cancer, e.g., pancreatic cancers, breast cancer, or head and neck squamosas cell cancer, and could provide valuable insights into the dynamics of the disease, as well as molecular characteristics [9,10,11].

The pretherapeutic influence of ctDNA has demonstrated a strong correlation between pre- and post-therapy levels, exemplified by patients undergoing treatments such as pembrolizumab with or without platinum/pemetrexed regimens for non-small-cell lung cancer. A statistically significant correlation was observed between the percentage alteration in ctDNA levels upon initial follow-up and the corresponding variation in tumor target lesions. Notably, reductions in ctDNA manifested pronounced associations with elevated rates of treatment response, prolonged median progression-free survival, and increased median overall survival relative to instances where ctDNA levels demonstrated increments [12,13,14]. Nevertheless, knowledge on dynamic changes in ctDNA during chemotherapy in mGEC is limited and there has been no universally established cut-off value for the clinical applicable use of liquid biopsy until now [15].

In this study, we aimed to test the prognostic impact of early dynamic changes in this novel biomarker and determine a cut-off for the prediction of treatment effects in restaging during the course of systemic chemotherapy for mGEC, which could eventually assist in early evaluation of response to treatment and allow for potential early changes in treatment in the future.

## 2. Materials and Methods

### 2.1. Patient Cohort

All patients in this study were treated at the Ordensklinikum Linz, an Austrian reference hospital for gastrointestinal cancer treatment, from December 2020 to January 2022. Patients were enrolled after histological confirmation of adenocarcinoma of the esophagus, gastroesophageal junction, or stomach and receipt of an interdisciplinary tumor conference decision for systemic treatment with palliative intent. This study was approved by the local ethics committee and informed consent was obtained from every patient included into the study prior to treatment initiation.

### 2.2. Sample Preparation

Liquid biopsy was collected from 37 patients with mGEC during clinical routine, before treatment initiation, and every two weeks thereafter, drawing 28.5 mL of blood per sample using cell-free DNA collection tubes (Roche, Basel, Switzerland) containing DNA stabilizers. The tubes were then centrifuged at 200 g for 10 min. Afterwards, the supernatant was transferred into new 15 mL tubes (Sarsted, Nümbrecht, Germany), followed by a second centrifugation for 10 min at 1500 g. The final product of 10 µL plasma was again transferred into new 15 µL tubes and stored at −20 °C until transportation from the study center to the Institute of Human Genetics (Medical University of Innsbruck, Innsbruck, Austria) for further analysis.

### 2.3. ctDNA Extraction

Preparation of cfDNA from plasma samples was performed by using the Chemagic 360 system and the CMG-1304 kit manufactured by Perkin Elmer (Waltham, MA, USA) following the manufacturer’s instruction. Therefore, 10 µL of prepared human plasma was collected from each study subject; 70 µL of the elution buffer, CMG-844 (Perkin Elmer, Waltham, MA, USA), was used to elute the DNA from the beads and resulted in a final DNA volume of 40–50 µL. The remaining liquid in the beads was considered residual. After the DNA elution step, the extracted cfDNA was quantified using the Quantus (Promega, Madison, WI, USA) fluorometer and stored at −20 °C until further use.

### 2.4. Individual Assay Design and Droplet Digital PCR (ddPCR)

Before ctDNA testing of the peripheral blood, histological specimens (either from pretherapeutic biopsy of the primary tumor or its metastases, or from the resected specimen in cases of metachronous metastases) were evaluated using the TruSight Tumor 170 (TST170) panel by Illumina Inc. (San Diego, CA, USA), which constitutes an enrichment-based targeted next-generation sequencing (NGS) platform tailored to examine the coding regions of 170 genes implicated in cancer. Utilizing DNA analysis via TST170 facilitates the identification of somatic variants encompassing single-nucleotide variants (SNVs), insertions/deletions (indels), and copy number variations (CNVs). Extensive validation has evidenced the efficacy of TST170 in molecularly profiling tumor tissues [16,17,18]. In this study, the QX200TM Droplet DigitalTM PCR System from Bio-Rad (Bio-Rad Laboratories, Hercules, CA, USA) was used to perform the screening for the most common KRAS alterations. Furthermore, a commercial multiplex screening kit provided by Bio-Rad Laboratories was utilized to screen all of the samples for variants in the respective gene detected by TST170. Single-mutation detection assays were requisitioned through the manufacturer’s exclusive online platform.

### 2.5. Statistics

Statistical analysis was performed in SPSS 26.0 (IBM, Coroporation, Armonk, NY, USA); Stata (version 18.5) (StataCorp LLC, College Station, TX, USA) and Microsoft Excel (version 16.80) (Microsoft Corporation, Redmond, WA, USA) on Windows were used for the visualization of graphs, drawings, and tables. Ordinal/nominal scaled data as well as non-normally distributed metric parameters were compared using the Mann–Whitney U test (e.g., sex), while normally distributed metric parameters (e.g., MAF) were compared using student’s *t*-test. A two-sided level of significance of *p* < 0.05 was applied to test statistical significance. The dynamics of ctDNA over time, reflecting changes in the Mutant Allele Frequency (MAF) for an individual, were characterized by computing the ratios of the MAF at various time intervals (i.e., after 2 or 4 weeks) and comparing them to their respective baseline values, set as the 100% reference. Area under the curve (AUC) analyses were conducted using the ROC analysis. The optimal cut-off in AUC analysis was determined by selecting the point nearest to the top-left corner of the plot, representing the best balance between sensitivity and specificity. Survival analyses, namely, progression-free survival (PFS) and overall survival (OS), were calculated using the Kaplan–Meier method.

## 3. Results

### 3.1. Patient Characteristic

**Figure 1:**
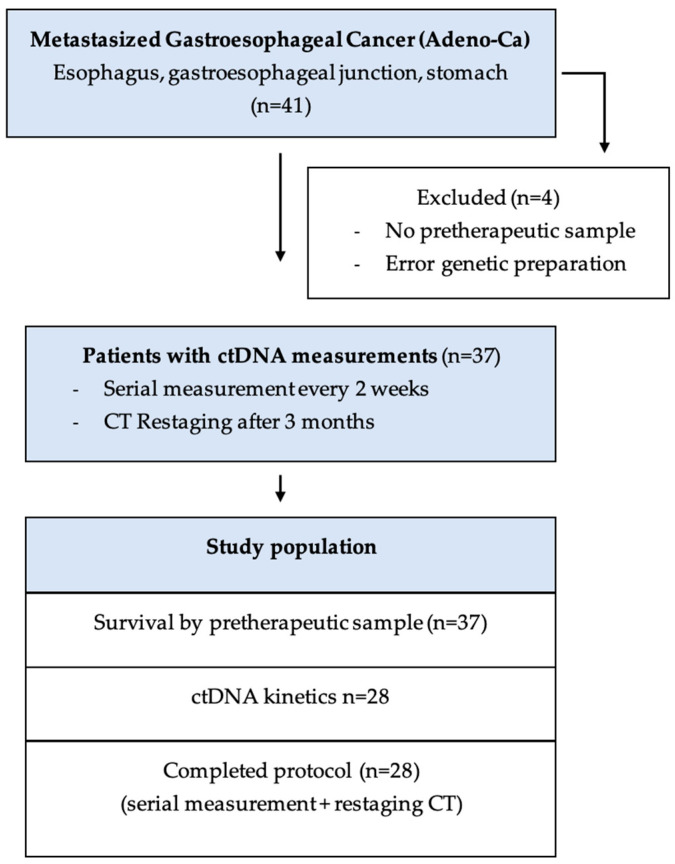
Consort flow diagram of study population.

A total of 41 patients with metastasized gastroesophageal adenocarcinoma (esophagus, gastroesophageal junction, and stomach) who received palliative chemotherapy at the Ordensklinikum Linz between December 2020 to January 2022 were included. However, four (9.7%) patients were excluded because no representative pretherapeutic genetic sample was gathered (see Figure 1).

A total of 78.1% of patients were male and the median age was 61.4 (IQR 47–81) years. The majority of the patients had a good performance status, with 77.8% being classified as ECOG 0 and only 3.3% having an ECOG status ≥ 2. The whole study population (n = 37) received palliative chemotherapy (62.2% 1st-line, 24.3% 2nd-line, 13.5% 3rd-line therapy or higher). A total of 13.5% of the study population underwent initial curative intended surgery, suffering from metachronous dissemination, whereas 81.1% had synchronous metastases. The anatomical tumor location was distributed as follows: esophagus (n = 13), esophagogastric junction (n = 4), and stomach (n = 20). The patients’ full characteristics are provided in Table 1, and there were no statistical differences within them regarding ctDNA detectability.

### 3.2. Mutational Pattern and Detection Rates

**Figure 2:**
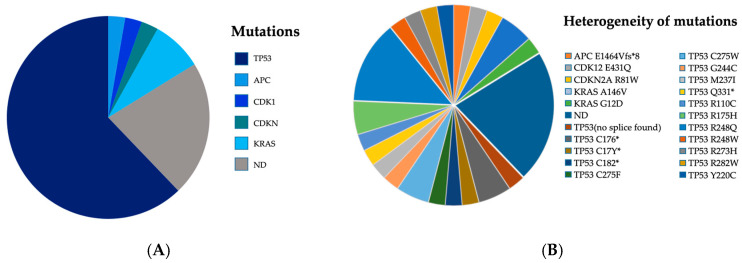
Mutational pattern of ctDNA targets (ddPCR) used after individual assay design from tumor tissue NGS given as genomic target families (**A**) and exact targets (**B**). Abbreviations: ND, not detectable.

Before ctDNA testing in the peripheral blood, histological specimens (either from pretherapeutic biopsy of the primary tumor or its metastases (83.8%), or from the resected specimen (16.2%)) were evaluated using the TruSight Tumor 170 (TST170) panel. After that, the peripheral blood was tested for the presence of the already known mutation of the primary tumor. ddPCR using individually designed assays showed a pretherapeutic detection rate of 77.8% in the peripheral blood when applying three mutant positive droplets as the threshold for positivity. The genetic work-up showed testable mutations of five major gene families in our study cohort. The majority of patients harbored TP53 (62.16%) followed by KRAS (8.11%) and minor percentages of APC (2.7%), CDK1 (2.7%), and CDKN (2.7%) mutations. A total of 22.2% of patients were regarded as ctDNA-negative (Figure 2A). TP53 R248Q mutation represents the highest frequency, observed in five instances, followed by TP53 R175H, TP53 C275W, KRAS A146V, and TP53 C176*, each occurring with a notable frequency. However, the exact work-up showed a very heterogenous distribution of 21 different targets (59.5%) for 37 patients (Figure 2B).

### 3.3. Response to Treatment Evaluation

**Figure 3:**
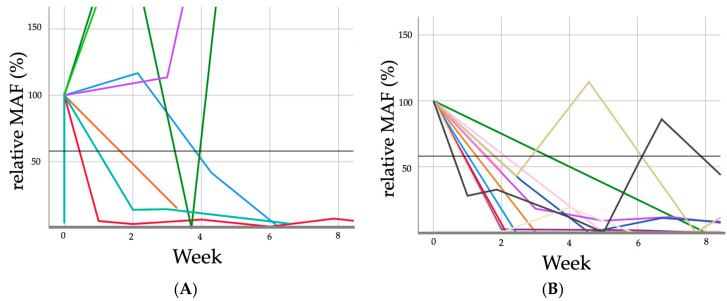
ctDNA kinetics stratified by response categories (each patients course is indicated as different color): Progressive disease (PD, (**A**)) and non-progressive disease (non-PD, (**B**)) with a cut-off line of 57.1%. Each patient’s ctDNA kinetics are represented using distinct colors. CtDNA refers to circulating tumor DNA; non-PD signifies non-progressive disease (including complete response, partial response, and stable disease). In addition to Figure 3, Table 2 has been included to provide a more comprehensive understanding of the data and graphical content. The table offers a detailed overview of the Mutant Allele Frequency (MAF) kinetics, categorized into two response groups: progressive disease and non-progressive disease. This addition allows for a more precise interpretation of the ctDNA dynamics presented in the figure and facilitates a deeper understanding of the results.; MAF denotes mutant allele frequency; PD indicates progressive disease.

**Figure 4:**
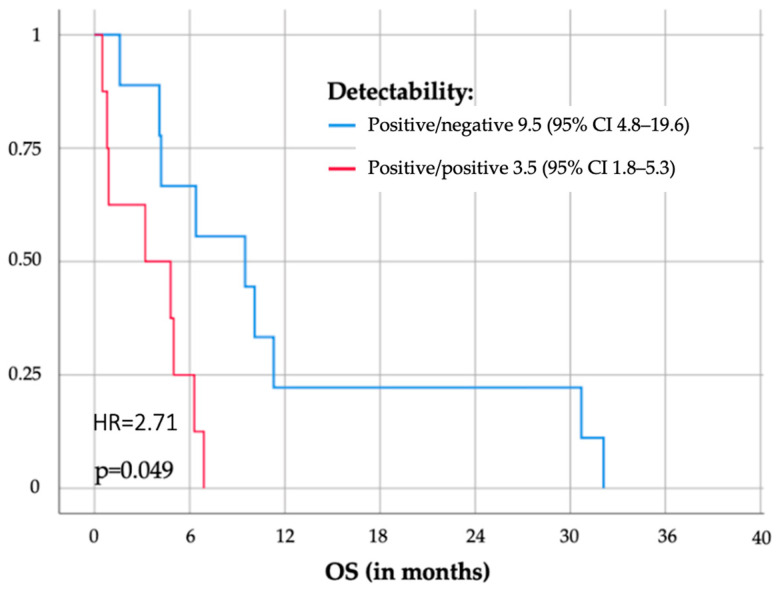
The impact of changes in ctDNA detectability (ctDNA pos/neg.) during treatment. Overall survival (OS) of 4 patients with mGEC stratified by their change in circulating tumor DNA (ctDNA) detectability. Patients starting as positive and becoming negative (blue) showed significantly better OS compared to patients starting as positive and staying positive during the whole course of palliative chemotherapy (red).

Patients experiencing progressive disease revealed notably different ctDNA curves in serial measurement during systemic treatment compared to patients who did not (partial response, stable disease, or complete remission), As illustrated in Figure 3 and Figure 4, as well as Table 2.

ROC analysis revealed an area under the curve (AUC) of 0.73 at a cut-off value of 57.1% for dynamic changes in circulating tumor DNA (at week 2) from baseline (given as 100%), with a sensitivity of 57.1% and a specificity of 90% for correctly predicting 76.5% of patients after only 2 weeks of treatment regarding radiological response to treatment at restaging after three months of systemic chemotherapy. Moreover, all of the patients with later non-progressive disease revealed after three months showed ctDNA dynamics declining to under 57.1% at the first measurable time point. Overall, response to treatment assessment was correct in 54.2% (pretherapeutically pos./neg.), 76.5% (dynamics at week 2), and 85% (dynamics at week 4), respectively.

### 3.4. Survival Analysis

**Figure 5:**
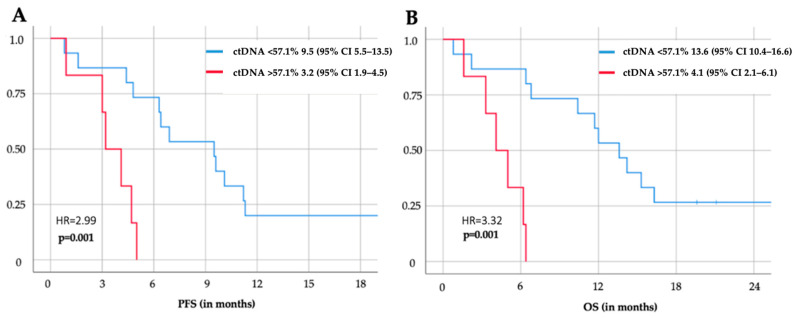
ctDNA dynamics after 2 weeks predict PFS (**A**) and OS (**B**). Applying a threshold for circulating tumor DNA (ctDNA) decline under the threshold of 57.1% of its pretherapeutic baseline after 2 weeks of systemic chemotherapy was associated with a significant prognostic impact on progression-free survival (**A**) and overall survival (**B**).

The mere detectability of pretherapeutic ctDNA in peripheral blood of patients with disseminated gastroesophageal cancer revealed no significant differences in OS (*p* = 0.563) and PFS (*p* = 0.787). However, the change in detectability during the course of the treatment (e.g., starting positive and turning negative vs. starting positive and staying positive) was of relevantly more prognostic relevance (9.5 vs. 3.5 months, *p* = 0.049). Moreover, patients who started treatment as positive and turned negative during the course of therapy (9.5 (95%Cl 4.8–19.6)) showed similar disease-free survival rates as patients who were regarded as ctDNA-negative from beforehand (8.4 (95%Cl 4.4–12.4)). Accordingly, patients who started negative but became ctDNA-positive during the course of the therapy (4.7 (95%Cl 4.7–4.7)) showed similar disease-free survival rates to patients who were regarded as positive and stayed positive (3.5 (95%Cl 1.8–5.3)) during the whole therapy (Figure 5).

Most importantly, a decline in ctDNA dynamics to the cut-off of 57.1% of the baseline value was significantly associated with OS (4.1 (95%Cl 2.1–6.1) vs. 13.6 (95%CI 10.4–16.6) months, *p* < 0.000) and PFS (3.2 (1.9–4.5) vs. 9.5 (95%CI 5.5–13.5) months, *p* = 0.001) after only two weeks of treatment (Figure 5).

## 4. Discussion

Liquid biopsy for prognostic purposes has been promoted recently due to advances in molecular biology techniques and next-generation sequencing technologies that have increased the sensitivity and accuracy of ctDNA testing while becoming more and more clinically applicable [19].

Historically, mutational analysis in cancer has predominantly relied on tumor tissue obtained through conventional biopsy. Although ctDNA has limitations, such as sample processing issues, assay specificity, and low shedding into the bloodstream by some tumor entities (e.g., pancreatic cancer), its analyses may provide more comprehensive information reflective of systemic tumor burden than the limited areas sampled by tissue biopsy; in addition, it has easy reproducibility and thus the possibility of non-invasive serial measurements [20]. However, the appliable testing strategy heavily depends on the tumor stage and entity, as for example the mutational spectrum of pancreatic cancer is very homogenous, with >90% of patients harboring KRAS mutations in tissue (eventually no need for prior tissue testing), while the mutational spectrum of colorectal and gastroesophageal cancer is very heterogenous [15]. Our findings affirm the broad range of potential targets for liquid biopsy in mGEC (Figure 3 and Table 2). Thus, tissue testing and individualized assay designing seems necessary for targeted small-spectrum ctDNA testing of the periphery in mGEC. Otherwise, broad-spectrum testing (e.g., NGS) is mandatory, but much more expensive when serial measurements are needed.

Recent research demonstrates that next-generation sequencing (NGS) is cost-effective as an oncology biomarker testing strategy under specific conditions. This evidence highlights the necessity for policy development that supports comprehensive evaluations of NGS, ensuring adequate reimbursement and access, and promoting its integration into routine clinical practice [21]. Nevertheless, multitarget assays still require improvement to achieve the necessary limits of detection for identifying low-frequency variants in ctDNA [21].

Besides that, the definition of ctDNA detectability itself is inconsistent in the literature and varies between different and even within the same modalities [22,23]. Among the available methods, from the perspective of price–performance ratio, ddPCR is the most sensitive approach for detecting ctDNA in cancer patients. On the other hand, compared to NGS, ddPCR has lower multiplexing capabilities. It may be beneficial to consider ways of enhancing the sensitivity of liquid biopsy as a prognostic biomarker. One potential avenue for exploration could be the introduction of priming agents that may help to reduce analyte clearance in vivo, thereby increasing diagnostic sensitivity [24,25]. However, the clinical applicable threshold could have to be adjusted then again as well.

Moreover, and in contrast to localized disease, in disseminated stages, pretherapeutic presence of ctDNA alone is not prognostic but rather indicates that the testing strategy is suitable, as all patients who bear mutations in tissue should be detectable in the periphery, as indicated by a systemic tumor burden. However, the dynamic change in ctDNA detectability during treatment is of more prognostic relevance (as shown in Figure 5). Even more prognostic relevance can be derived by evaluating ctDNA dynamics at a specific cut-off (i.e., a decline to under 57.1% of the baseline value like suggested in this study for the first time) after 2 or 4 weeks post-treatment initiation. These findings underscore the potential of ctDNA dynamics as a promising indicator for treatment response monitoring in mGEC patients, offering insights into the effectiveness of systemic therapies and informing clinical decision-making processes. However, this study represents a pioneering effort in evaluating the role of ctDNA kinetics as a prognostic tool in mGEC. As the first study of its kind to explore this specific topic, it provides a novel approach to treatment monitoring and early response evaluation, potentially shifting clinical practice. Given the small sample size, the results are intended to serve as a hypothesis-generating foundation rather than definitive conclusions. This research lays the groundwork for future large-scale multicenter studies, which are essential for validating these findings and refining the clinical applicability of ctDNA dynamics in mGEC. The promising initial results suggest that ctDNA kinetics could become a valuable tool for predicting treatment response and guiding therapy adjustments, but larger cohorts with more diverse populations are needed to confirm these findings and establish universally applicable thresholds. Moreover, ctDNA dynamics may even allow for early changes in treatment in the future. Although the cut-off revealed in this study was already of statistical prognostic relevance and showed promising accuracy in predicting response to treatment, it needs to be re-evaluated by bigger cohorts in the future to allow for the deduction of a clinically appliable and universal threshold for actual changes in treatment in the future. However, using a decline to under approximately 60% of the baseline to determine if tumor markers are of prognostic relevance is not new and seems legitimate, as it was recently observed in other tumor entities and stages as well (e.g., CA 19-9 in locally advanced pancreatic cancer). Similar thresholds for ctDNA dynamics were also significantly associated with longer OS and PFS for colorectal and pancreatic cancer at metastasized stage [26].

Not only did the different applications for liquid biopsy assessment differ in their ability to predict response to treatment, but they also differed in their immediate prognostic relevance (OS, PFS). Whereas mere detectability of pretherapeutic ctDNA revealed no significant differences in OS and PFS (*p* = 0.563/0.787) in mGEC, the change in detectability during the course of the treatment (e.g., starting as positive and turning negative vs. starting as positive and staying positive) was of immense prognostic relevance (12.2 vs. 3.5 months, *p* = 0.049). This effect was even surpassed by the testing strategy at the novel threshold presented in this study (*p* < 0.001) but remains hypothesis-generating and needs further validation in bigger sample sizes. Nevertheless, both liquid biopsy applications have not been described for mGEC yet.

This makes perfect sense, as we should be able to detect tumor-specific fragments in the blood of every patient with systemic disease when using an ideal testing regimen, thereby minimizing the potential impact of pretherapeutic positivity on survival outcomes. In contrast, it is apparent but pertinent to note that in contrast, in localized disease stages, peripheral detection of ctDNA is expected to have great influence DFS and OS. Nonetheless, insufficient sensitivity may contribute to a limitation in the actual clinical applicability of ctDNA, as false-negative results in particular may even prevent or delay appropriate treatment (if, e.g., used as indicators to stop or de-escalate treatment). Thus, ctDNA detection should be seen as potential complementation to the current clinical gold standard if positive (displaying high tumor burden) or increasing (increasing tumor burden, reflecting inefficient treatment, and thus allowing for the chance of early changes in treatment).

The sensitivity issue could be addressed in the future by optimizing methods for cfDNA extraction, such as using more sensitive or specialized kits that reduce the loss of ctDNA and increase yield (collection tubes with DNA stabilizers were used in this study), apart from the higher DNA input for analysis (ddPCR in this study). However, despite the good sensitivity of ddPCR for specific mutations known from prior tissue analysis, multiplexed target panels could expand the range of genetic mutations analyzed, especially using more comprehensive NGS panels. Detecting a broader spectrum of ctDNA variants combined with more sensitive techniques could increase detection rates across heterogeneous tumor types, although this would significantly increase costs. Additionally, our study incorporated longitudinal analysis of ctDNA levels during treatment, displaying improved sensitivity and prognostic impacts. By tracking changes in ctDNA over time, minor fluctuations may be detected, indicating treatment effects more quickly and reliably. Furthermore, a combination of ctDNA testing with other already established biomarkers or using a score might enhance the overall sensitivity.

However, larger scale studies might evaluate ways to refine the thresholds for detecting ctDNA positivity or defining the sensitivity cut-offs based on a more granular analysis of ctDNA dynamics and mutation load in order to potentially identify low-frequency mutations that are clinically relevant.

Historically, survival rates in clinical trials for this patient population have been less than one year [27]. Recent advancements, e.g., Keynote 590 and Keynote 811 trials, have shown that the combination of immune checkpoint inhibitors (ICIs) with CTX has led to improved survival outcomes for patients with esophageal cancer [28,29,30]. A persistent challenge in clinical practice is the prediction of individual susceptibility to side effects, which is crucial for making informed decisions regarding treatment modifications [31]. ctDNA-guided treatment has the potential to reduce chemotoxicity and offers the possibility to escalate or de-escalate systemic treatment in the future (Figure 5), so it may find its place in gastrointestinal cancers irrespective of tumor stage.

In subsequent clinical applications, ctDNA may serve as a determinant for transitioning to second-line chemotherapy. As previously demonstrated by Shen et al. and Kojima et al., tislelizumab, nivolumab, or pembrolizumab as second-line therapy exhibited prolonged overall survival compared to conventional chemotherapy, accompanied by a decreased occurrence of treatment-related adverse events [32,33,34]. The paucity of scientific papers poses a significant challenge in establishing ctDNA as a reliable predictor for chemotherapy switches in clinical practice. Further research is warranted to elucidate the role of ctDNA in guiding therapeutic decisions and optimizing treatment strategies for cancer patients. At this stage, we can only refer to studies from other organ entities (e.g., colorectal or pancreatic cancer), as there are very limited data on mGEC, such as the DYNAMIC trial where using ctDNA as a guiding strategy for the management of stage II colon cancer resulted in a reduction in the administration of adjuvant chemotherapy while maintaining recurrence-free survival rates [35,36]. The prognostic value of liquid biopsy-guided palliative systemic chemotherapy has not been proven yet, but our study provides promising prospects for ctDNA-guided changes in treatment as a method that can be easily applied in addition to routine staging for mGEC, showing a prognostic benefit 80% faster than current gold standard restaging (CT) for the first time in this tumor entity. However, this needs to be validated in larger scale multicentric randomized studies in order to provide a robust threshold and allow for actual changes in treatment based on liquid biopsy results in the future.

### Limitations

We are aware of several limitations of this explorative pioneer study that should be acknowledged in the interpretation of its findings. Besides the monocentric observational study design, the small cohort may restrict the generalizability of our results and did not allow for subanalysis of, for example, different treatment lines. Moreover, the ROC’s reliability is compromised due to the small sample size, which limits the statistical power of our findings (also regarding the power of the respective definitive threshold for prognosis prediction by ctDNA kinetics). Furthermore, the multivariate survival analysis provided no statistically significant co-variates in this limited patient pool. Thus, potential co-variates need to be evaluated in larger future studies. Despite these limitations, the results provide a preliminary indication that warrants further investigation by larger cohorts in order to assess a clinically applicable threshold for potential early changes in treatment in the future. However, the prognostic impacts of peritherapeutic changes in ctDNA seem to be substantial enough to even find statistical differences in small cohorts. Nevertheless, larger scale multicentric randomized studies need to prove our conclusions in the future.

Moreover, future studies should emphasize the definitive benefit of clinically applicable and cost-effective liquid biopsy-guided treatment approaches in the evaluation of interventions, such as early treatment changes against standard-of-care practices.

Nonetheless, insufficient sensitivity may contribute to a limitation in the actual clinical applicability of ctDNA, as false-negative results in particular may even prevent or delay appropriate treatment (if, e.g., used as indicators to stop or de-escalate treatment). Thus, ctDNA detection should be seen as potential complementation to the current clinical gold standard if positive (displaying high tumor burden) or increasing (increasing tumor burden, reflecting inefficient treatment, and thus allowing for the chance of early changes in treatment).

However, as this technology advances, its limitations should also be acknowledged. For instance, the sensitivity of ctDNA detection may vary depending on tumor type and stage, and false negatives may still occur. Additionally, standardized thresholds for ctDNA changes need to be refined through large-scale studies to ensure reproducibility across different patient populations and clinical settings. Furthermore, more research is needed to assess the cost-effectiveness and feasibility of implementing ctDNA testing as part of routine clinical workflows.

## 5. Conclusions

This study underscores the potential of ctDNA to significantly impact the standard of care for mGEC patients. Moreover, and to the best of our knowledge, for the first time, this study provides a prognostic cut-off after only 2 weeks of systemic treatment for an easily implementable, cost-effective, and thus clinically applicable testing regimen in this tumor entity. CtDNA not only facilitates early detection but may also enable timely therapeutic interventions in the future. It is essential to recognize that ctDNA should not be relied upon as a sole diagnostic or prognostic tool for disease progression (as there is a strong correlation with tumor burden, PFS, and OS); rather, it could complement existing diagnostic methods, aiding in early detection of disease progression and thus guiding potential therapy adjustments in, e.g., palliative chemotherapy after only two weeks of treatment (80% faster than the current gold standard CT), with a sensitivity of 57.1% and a specificity of 90% for correct restaging assessment, as shown in this study. For patients, this approach holds promise for minimizing unevaluated chemotherapy-related toxicity and may improve survival through timely therapy modifications. However, further investigations involving larger cohorts are warranted to validate this prognostic impact, besides potentially elaborating on the sensitivity issue of the current testing strategy.

## Figures and Tables

**Table 1:** Demographics for patients with metastasized gastroesophageal cancer.

mGEC	Overall		ctDNA pos.		ctDNA neg.		*p*
	n = 37		n = 21		n = 16		
Age	61	(47–81)	62	(51–78)	61	(51–80)	0.380
Sex							0.430
Male	32	(78.05%)	6	(100)	11	(68.75)	
Female	5	(21.95%)	0		4	(31.25)	
ECOG PS							0.510
0	29	(77.8%)	16	(76.2)	13	(81.3)	
1	7	(18.9)	4	(19.0)	3	(18.7)	
≥2	1	(3.3)	1	(4.8)			
CTX Line							0.869
1st Line	23	(62.2%)	13	(61.9)	10	(62.5)	
2nd Line	9	(24.3%)	6	(28.6)	3	(18.8)	
≥3rd Line	5	(13.5%)	2	(9.6)	3	(18.8)	
T-stadium							0.497
cTx	14	(37.8%)	9	(42.9)	5	(31.3)	
cT1							
cT2	3	(8.1%)	2	(9.5)	1	(6.3)	
cT3	17	(45.9%)	8	(38.1)	9	(56.3)	
cT4	3	(8.1%)	2	(9.5)	1	(6.3)	
N-stadium							0.871
0	5	(13.5%)	2	(40)	3	(60)	
1	15	(40.5%)	2	(15.9)	13	(84.1)	
2	4	(10.8%)	1	(25)	3	(75)	
3	2	(5.4%)	0	(0)	2	(100)	
+	2	(24.4%)	0	(0)	2	(100)	
x	9	(5.4)	2	(22.2)	7	(77.8)	
Synchronous	32	(83.8%)	18	(85.7)	13	(81.3)	0.724
Metachronous	6	(16.2%)	3	(14.3)	3	(18.3)	0.258

All values are given as numbers (%) unless otherwise indicated. Abbreviations: ctDNA—circulating tumor DNA; ECOG PS—Eastern Cooperative Oncology Group performance state; CTX—chemotherapy; T-Stadium—primary tumor stage. N-stadium indicates spread to nearby lymph nodes.

**Table 2:** MAF-kinetics based on two response categories: progressive disease (A) and non-progressive disease (B). MAF denotes mutant allele frequency.

(**A**)			
**Initial Relative MAF in %**	**Relative MAF After 2 Weeks in %**	**Relative MAF After 4 Weeks in %**	**Relative MAF After 6 Weeks in %**
100	113.0	346.9	1034.3
100	116.7	51.7	7.0
100	240	59.7	470
100	45	0.0	0
100	200	17	0
100	5.3	11.7	0.3
100	0	0	0
100	18.1	11.8	9.0
(**B**)			
**Initial relative MAF in %**	**Relative MAF after 2 Weeks in %**	**Relative MAF after 4 Weeks in %**	**Relative MAF after 6 Weeks in %**
100	2.4	2.1	1.7
100	0	19.1	0
100	0	0	0.4
100	5.3	3.0	6.4
100	0	0	0
100	18.1	9.0	11.8
100	14.9	0	0
100	39.7	0	11.0
100	46.2	12.4	13.0
100	42.9	114.3	0
100	13.8	14.2	3.3
100	42.9	35.7	0
100	0.3	0.2	0.4

## Data Availability

The original data are available on reasonable request.
